# Risk factors and a predictive model for nonfilter-associated inferior vena cava thrombosis in patients with lower extremity deep vein thrombosis

**DOI:** 10.3389/fcvm.2022.1083152

**Published:** 2023-01-11

**Authors:** Maofeng Gong, Jie Kong, Yadong Shi, Boxiang Zhao, Zhengli Liu, Xu He, Jianping Gu

**Affiliations:** Department of Interventional and Vascular Radiology, Nanjing First Hospital, Nanjing Medical University, Nanjing, Jiangsu, China

**Keywords:** inferior vena cava thrombosis, vena cava filter, incidence, risk factors, deep vein thrombosis

## Abstract

**Objective:**

Nonfilter-associated inferior vena cava thrombosis (IVCT) is an under-recognized but severe state of venous thromboembolism. The aims of this study were to investigate risk factors and develop a prediction model based on clinical data and imaging findings to evaluate the probability of IVCT in patients with lower extremity deep vein thrombosis (LEDVT).

**Methods:**

A single-center retrospective cohort study was conducted. We analyzed the clinical data and multimodal imaging findings of consecutive patients with confirmed LEDVT between February 2016 and January 2022. The demographics, presentation of LEDVT, laboratory examination, thrombus characteristics, comorbidities and risk factors for LEDVT, and imaging findings were analyzed using an independent *t*-test, *Chi-square* test, *Fisher's* exact test, and regression analysis to determine the univariable and multivariable associations and to establish a predictive model to assess the probability of IVCT.

**Results:**

A total of 267 eligible patients were included, of whom 40 were in the IVCT group and 227 were in the non-IVCT group. The incidence of nonfilter-associated IVCT was 15.0% (40/267). Age < 63.5 years [odds ratio (OR) 2.54; 95% confidence interval (CI), 1.10–5.85, *p* = 0.029], male sex (OR 2.82; 95% CI, 1.19–6.72, *p* = 0.019), proximal DVT (OR 8.21; 95% CI, 1.01–66.76, *p* = 0.049), bilateral DVT (OR 7.30; 95% CI, 3.28–16.21, *p* < 0.001), and D-dimer >4.72 μg/ml (OR 4.64; 95% CI, 1.80–11.72, *p* = 0.001) were risk factors for IVCT's occurrence. Then, we established a prediction model based on these risk factors. The diagnostic efficiency [area under the curve (AUC) of receiver operating characteristic (ROC) curve was 0.858] for predicting IVCT was superior to that of isolated risk factors, including age < 63.5 years (AUC of ROC curve was 0.624) or D-dimer >4.72 μg/ml (AUC of ROC curve was 0.656).

**Conclusion:**

Age < 63.5 years, male sex, proximal LEDVT, bilateral LEDVT and D-dimer >4.72 μg/ml were risk factors. The diagnostic efficiency of the predictive model for predicting IVCT was superior to that of a single risk factor alone. It may be used for predicting the probability of nonfilter-associated IVCT in patients with LEDVT.

## Introduction

Inferior vena cava thrombosis (IVCT) is an under-recognized but severe state of venous thromboembolism (VTE) that is associated with significant morbidity and mortality (1, 2). The major predisposing factor for IVCT is the long-term implantation of inferior vena cava (IVC) filters, which is estimated to have an increasing incidence ranging from 5 to 30% due to the exponential use of unretrieved filters (2–4). Moreover, IVCT can also be present in patients without IVC filters (2). It is estimated that the incidence of nonfilter-associated IVCT is 4–15% among patients with confirmed lower extremity deep vein thrombosis (LEDVT) (4, 5). However, the true incidence may be underestimated due to the lack of standardized approaches for its detection (1, 2) and underreporting (4). Of note, compared with filter-associated IVCT, nonfilter-associated IVCT may be more prone to lead to life-threatening conditions, with twice the mortality rate than thrombosis confined in the filters (2, 6–8). The genesis is likely to lurk in a higher incidence of fatal pulmonary embolism (PE), which serves as a severe consequence of clot migration (9).

Despite the findings mentioned above, there is currently an overall paucity of specific guidelines within the literature to aid with the available screening when suspected IVCT occurs. The IVCT clinical signs and symptoms are insidious and non-specific and are frequently concealed and confused in LEDVT. Hence, the detection of nonfilter-associated IVCT depending on clinical features alone is challenging (10), and alternative screening programs for IVCT remain ambiguous (2, 10, 11). Dedicated duplex ultrasonography is usually operator dependent, and visualization of IVCT is often hampered due to bowel gas or obesity (8, 12). Computed tomography (CT), magnetic resonance imaging (MRI), and transcatheter venography can overcome this inherent limitation (8). However, the majority of LEDVT patients do not have IVCT. Hence, screening all suspected or inapparent patients with LEDVT routinely with CT and/or MRI for the diagnosis of IVCT can be tedious, low-yield, and cost-ineffective (1–3). However, considering the potential severe risks (7, 9, 11), it seems to be more of a help than a hindrance to screen in selected LEDVT patients with high-risk factors for IVCT. However, the risk factors for IVCT have not been well elucidated.

The aim of this study was to evaluate the risk factors for nonfilter-associated IVCT, as well as to establish a predictive model for LEDVT patients, which is expected to help develop an individualized screening plan, thus avoiding the unnecessary economic burden of CT examination and the later risk of radiation- and contrast medium-induced nephropathy.

## Methods

### Patients and study design

**Figure 1:**
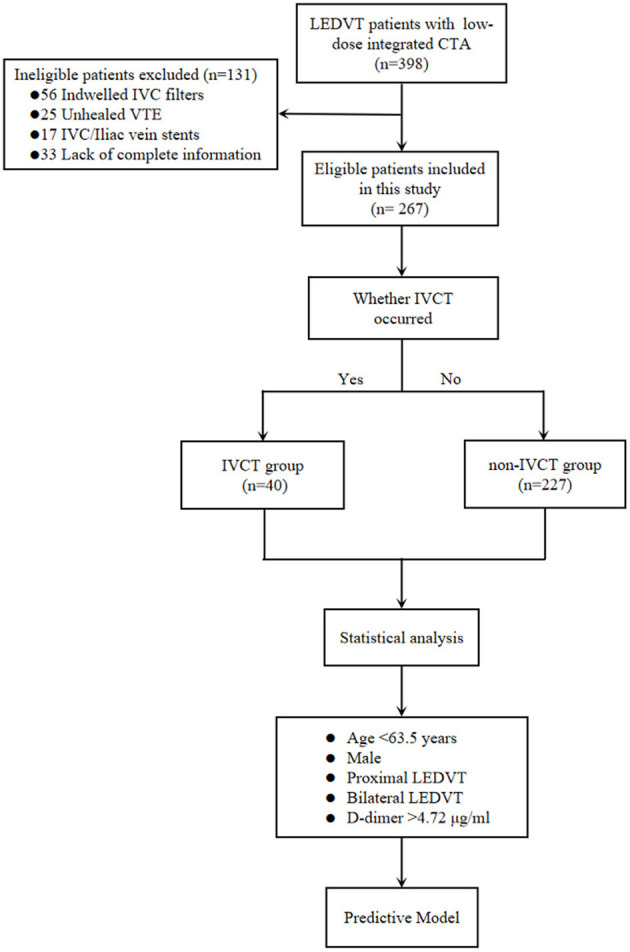
The study flowchart of the inclusion and exclusion criteria of LEDVT patients and the predictive model-building process. LEDVT, lower extremity deep vein thrombosis; CTA, computed tomography angiography; IVC, inferior vena cava; VTE, venous thromboembolism; IVCT, inferior vena cava thrombosis; DVT, deep vein thrombosis.

The data collection protocol and informed consent were approved by our Institutional Review Board. This was a retrospective cohort study including consecutive confirmed LEDVT patients at a single academic center from February 24, 2016, to January 18, 2022. The inclusion criteria were deep vein thrombosis (DVT) patients with/without PE who had complete clinical data and who underwent low-dose integrated CT angiography (combined CT angiography of the pulmonary artery, IVC and lower limb veins) within 48 h (h) after their admission. Exclusion criteria were previous indwelling IVC filters and IVC/iliac vein stents, unhealed VTE or incomplete data. The data were retrospectively obtained from the medical database system and/or the paper records to identify eligible patients. The baseline demographics (mainly comprising the age and sex), onset of symptoms at presentation, hematological examination (mainly including D-dimer levels) within the first 24 h, whether coupled with PE, thrombus limbs [bilateral limbs or unilateral limb (left or right side)], DVT segment [proximal DVT or isolated distal deep vein thrombosis (IDDVT)], external compression of IVC, comorbidities [mainly containing hypertension, diabetes mellitus, coronary artery disease (CAD), history of cerebral vascular disease and peripheral artery disease (PAD)] and thrombotic risk factors [incorporates trauma, major surgery history, immobilization, cancer, May–Thurner syndrome, thrombophilia, and previous VTE (has been cured)] were all analyzed. A total of 398 potentially eligible patients with identified LEDVT were collected, and 131 patients were subsequently excluded because of indwelling IVC filters (*n* = 56), unhealed VTE (*n* = 25), IVC/iliac vein stents (*n* = 17) or lack of complete data (*n* = 33) (the study flowchart is shown in Figure 1). Of the remaining 267 included patients, 40 patients with IVCT were divided into the IVCT group, and 227 were divided into the non-IVCT group.

### Diagnostic methods and details

The initial diagnosis of LEDVT was determined by medical history and physical examination, which was then verified by ultrasound doctors with duplex ultrasonography [including the iliac, femoral, popliteal, and distal veins (including anterior tibial, posterior tibial, peroneal, gastrocnemius muscle, and soleus muscle veins)]. Additionally, plasma D-dimer was tested within 24 h after admission using a quantitative enzyme-linked immunosorbent assay provided by the central laboratory of the academic hospital (7). A value > 500 ng/ml was deemed abnormal for patients aged < 50 years, and an age-adjusted threshold (age × 10 ng/ml) was used for patients aged > 50 years (1, 2). A low-dose integrated CT (128-slice dual source CT, SOMATOM Definition Flash, Siemens, Germany) angiography (including a combined CT angiography of pulmonary artery, IVC and lower extremity veins) was objectively performed within 48 h after initial diagnosis to confirm the distribution and extent of thrombosis. The interpretation of the low-dose integrated CT angiography was based on the initial radiologist's reading. The IVCT was defined as the existing filling defect in the IVC at the venous phase of CT angiography. The diagnosis was initially performed by radiologists *via* CT angiography and was ultimately confirmed by two independent reviewers (ZBX and HX). Proximal LEDVT included thrombi in the common iliac vein, external iliac vein, common femoral vein, proximal and distal segments of the femoral vein, and/or popliteal vein, and IDDVT included thrombi in distal veins, including the anterior tibial vein, posterior tibial vein, peroneal vein, gastrocnemius muscle vein, and soleus muscle vein.

### Statistical analysis

Statistical analyses were performed by *SPSS* statistical software package (version 23.0; *SPSS* statistical software, Chicago, Illinois, USA) and R statistical language software (version 3.6.3; R Foundation). Continuous data are presented as the mean ± standard deviation (SD), and categorical data are given as counts (percentage). When assessing the correlation between the two groups and comparing continuous data, including age, onset of symptoms at presentation, and D-dimer value, a Student's *t-*test was used. The significance of categorical data was tested with a *chi-square* test or *Fisher's* exact test. The predictive factors for IVCT were assessed with logistic regression; the univariate approach was followed by multivariate analyses. The predictive power of age and D-dimer values as a suspected diagnosis and the predictive ability of this study were evaluated with receiver operating characteristic (ROC) curves using R software. The visualization of multivariate results was performed with GraphPad Prism (GraphPad Prism Software 9.0, San Diego, California). A risk score was developed based on regression coefficients from the final multivariate model. Calibration and discrimination of the logistic regression model were assessed by the Hosmer–Lemeshow goodness of fit (GOF) test, and the area under the curve (AUC) was calculated. DeLong's test was used to compare the difference in calibration and discrimination of the logistic regression between a single factor and the predictive model. Findings with a *p* < 0.05 were deemed statistically significant.

## Results

### Baseline demographics and characteristics of patients with/without IVCT

Among the 267 eligible patients in this study, 15.0% (40/267) of patients with IVCT were divided into the IVCT group, and 85% (227/267) were divided into the non-IVCT group. The mean age of these included patients was 60.58 ± 15.56 years old, and 52.4% (140/267) were male. The mean time of symptom onset at presentation was 10.59 ± 10.67 days. The main laboratory examination of the D-dimer value was 9.13 ± 11.29 μg/ml. In the same CT angiography examination session, PEs were detected in 55.4% (148/267) of these patients. The main, lobar, and segmental pulmonary arteries were affected in 18.9 (28/148), 43.2 (64/148) and 37.8% (56/148) of these patients, respectively. Regarding limb thrombi, 73.8% (197/267) of these patients suffered from unilateral LEDVT, of whom left-side LEDVT accounted for 63.4% (125/197). For DVT segments, 78.7% (210/267) of these patients experienced proximal LEDVT. The external compression of the IVCT in patients with IVCT was slightly higher than that in those without (*p* = 0.085). The leading comorbidities of these patients were hypertension (39.7%) and diabetes mellitus (18.0%), and the major risk factors were May–Thurner syndrome and immobilization, which were present in 29.2 (78/267) and 21.3% (57/267) of these patients, respectively. The univariable associations of the demographics, presentation, laboratory examination, thrombus characteristics, comorbidities and risk factors for these patients are summarized in Table 1, showing that compared to the patients with non-IVCT, the factors that predicted the probability of IVCT were as follows: age (*p* = 0.021), gender (*p* = 0.006), median D-dimer value (*p* = 0.046), thrombus limbs (*p* < 0.001), and DVT segments (*p* = 0.002).

**Table 1:** Demographics, presentation, lesion characteristics, cancer conditions, comorbidities and risk factors of IVCT patients.

**Characteristic**	**IVCT group (*n* = 40)**	**Non-IVCT group (*n* = 227)**	***p-*value**
Age, years, mean ± SD	55.35 ± 14.85	61.51 ± 15.52	0.021
Age < 63.5 years, *n* (%)	28 (70.0)	102 (44.9)	0.003
Age > 63.5 years, *n* (%)	12 (30.0)	125 (55.1)	
**Gender**, ***n*** **(%)**			
Male	29 (72.5)	111 (48.9)	0.006
Female	11 (27.5)	116 (51.1)	
Onset of symptoms at presentation, *n* (%)	9.65 ± 8.85	10.76 ± 10.97	0.547
Median D-dimer value (μg/ml)	12.41 ± 11.58	8.56 ± 11.17	0.046
D-dimer >4.72 μg/ml	31 (77.5)	112 (49.3)	0.001
D-dimer < 4.72 μg/ml	9 (22.5)	115 (50.7)	
LEDVT coupled with PE, *n* (%)	25 (62.5)	123 (54.2)	0.329
Main	8 (32.0)	20 (16.3)	0.067
Lobar	11 (44.0)	53 (43.1)	0.933
Segmental	6 (24.0)	50 (40.7)	0.118
**Thrombus limbs**, ***n*** **(%)**			
Bilateral limbs, *n* (%)	25 (62.5)	44 (19.4)	< 0.001
Unilateral limb, *n* (%)	14 (35.0)	183 (80.6)	
Left side	10 (71.4)	115 (62.8)	0.520
Right side	4 (28.6)	68 (37.2)	
**LEDVT level**, ***n*** **(%)**			
Proximal LEDVT	39 (97.5)	171 (75.3)	0.002
IDDVT	1 (2.5)	56 (24.7)	
**Comorbidities**, ***n*** **(%)**			
Hypertension	14 (35.0)	92 (40.5)	0.510
Diabetes mellitus	7 (17.5)	41 (18.1)	0.932
CAD	1 (2.5)	22 (9.7)	0.135
History of cerebral vascular disease	2 (5.0)	33 (14.5)	0.099
PAD	3 (7.5)	20 (8.8)	0.785
**Risk factors**, ***n*** **(%)**			
Trauma	2 (5.0)	23 (10.1)	0.304
Major surgery history	5 (12.5)	35 (15.4)	0.633
Immobilization	6 (15.0)	51 (22.5)	0.760
Cancer	4 (10.0)	21 (9.3)	0.881
May–Thurner syndrome	10 (25.0)	68 (30.0)	0.525
Thrombophilia	3 (7.5)	18 (7.9)	0.926
Previous VTE	12 (30)	43 (18.9)	0.111
External compression of IVC	5 (12.5)	12 (5.3)	0.085

IVCT, inferior vena cava thrombosis; LEDVT, lower extremity deep vein thrombosis; PE, pulmonary embolism; ID DVT, isolated distal deep vein thrombosis; IVC, inferior vena cave; CAD, coronary artery disease; PAD, peripheral artery disease; VTE, venous thromboembolism.

Continuous data are presented as the means ± standard deviations; categorical data are given as the counts (percentage).

### Predictive risk factors of age, gender and D-dimer for IVCT

**Figure 2:**
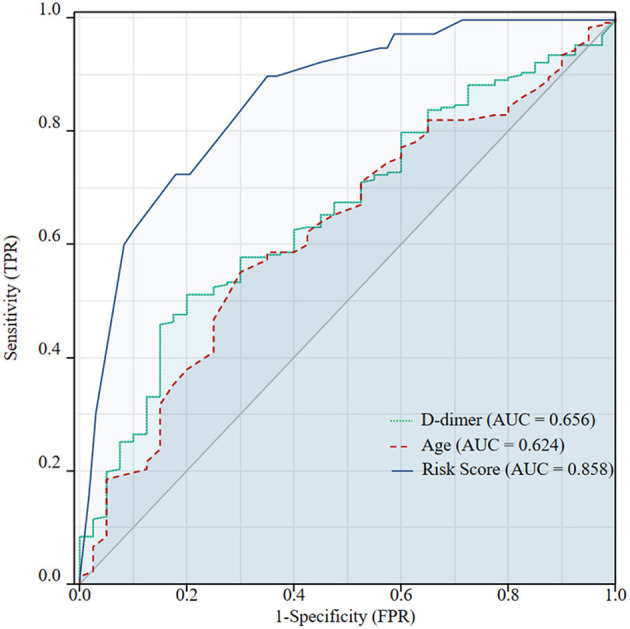
Receiver operating characteristic (ROC) curves were used to identify the optimal cutoff values of age and D-dimer for inferior vena cava thrombosis (IVCT) in patients with lower extremity deep vein thrombosis (DVT). The cutoff values were 63.5 years and 4.72 μg/ml, respectively. The AUC of the ROC for age < 63.5 years was 0.624 (95% CI, 0.533–0.715) and 0.656 (95%CI, 0.571–0.742) for a D-dimer value >4.72 μg/ml. Predicted probabilities of IVCT according to the combined five features: age < 63.5 years, male sex, proximal DVT, bilateral DVT, and D-dimer >4.72 μg/ml. The AUC of the ROC was 0.858 (95% CI, 0.799–0.916). The diagnostic efficiency of this multivariable model was significantly better than a single risk factor of age < 63.5 years (*p* < 0.001) or D-dimer >4.72 μg/ml (*p* < 0.001) alone.

The mean age of the included patients with IVCT was 55.35 ± 14.85 years old, which was younger than the 61.51 ± 15.52 years in patients with non-IVCT (*p* = 0.021), and the D-dimer levels in the IVCT group were significantly higher than those in the non-IVCT group (12.41 ± 11.58 vs. 8.56 ± 11.17 μg/ml, *p* = 0.001). After univariable analysis, the predictive values of age and D-dimer were analyzed by ROC curves to identify the optimal cutoff values. Age < 63.5 years and D-dimer value >4.72 μg/ml were discriminant [AUC = 0.624 (95% CI, 0.533–0.715) and AUC = 0.656 (95% CI, 0.571–0.742), both *p* < 0.05, respectively] for predicting IVCT (ROC curves are shown in Figure 2). The indicators of age < 63.5 years and D-dimer value >4.72 had sensitivities of 55.1 and 51.1% and specificities of 70.0 and 80.0%, respectively. The positive predictive values for age < 63.5 years and D-dimer >4.72 were 91.2 and 93.5%, respectively, and the negative predictive value was 21.5 and 22.4%. Therefore, more patients with age < 63.5 years and D-dimer >4.72 μg/ml developed IVCT than patients with age > 63.5 years (70.0 vs. 44.9%, *p* = 0.003) and D-dimer < 4.72 μg/ml (77.5 vs. 49.3%, *p* = 0.001). Except as mentioned above, there were more male patients in the IVCT group than in the non-IVCT group (72.5 vs. 48.9%, *p* = 0.006).

### The incidence and relationship between thrombus distribution and IVCT

The overall incidence of nonfilter-associated IVCT among LEDVT patients in this study was 15.0% (40/267). Of note, included patients encountered more proximal DVT and fewer IDDVT in the IVCT group compared with the non-IVCT group (97.3 vs. 75.3 and 2.5 vs. 24.7%, *p* = 0.002). In addition, thrombus limbs in IVCT group focused on bilateral LEDVT (62.5 vs. 19.4%) compared with more unilateral LEDVT (80.6 vs. 35.0%) that occurred in the non-IVCT group (*p* < 0.001), but the difference between the left side limb and right side limb was not statistically significant (*p* = 0.520).

### Predictive models for nonfilter-associated IVCT

**Figure 3:**
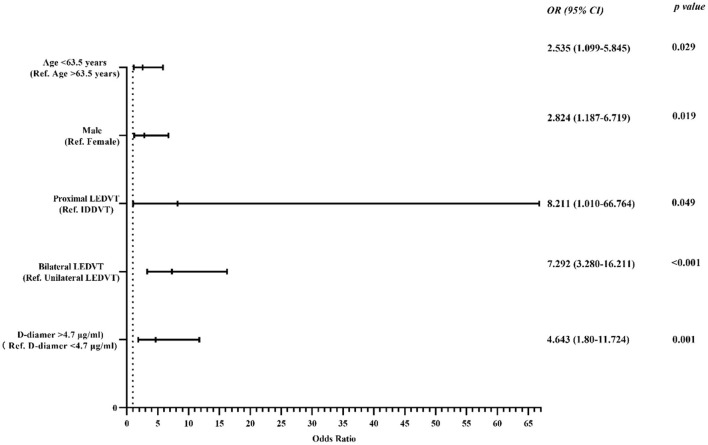
Forest plot used to visualize the multivariate regression analysis of risk factors for IVCT in lower extremity DVT patients. IVCT, inferior vena cava thrombosis; LEDVT, lower extremity deep vein thrombosis; IDDVT, isolated distal deep vein thrombosis; Ref., reference.

Multivariate logistic regression analyses showed that age < 63.5 years [odds ratio (OR) 2.54; 95% confidence interval (CI), 1.10–5.85, *p* = 0.029], male sex (OR 2.82; 95% CI, 1.19–6.72, *p* = 0.019), proximal DVT (OR 8.21; 95% CI, 1.01–66.76, *p* = 0.049), bilateral DVT (OR 7.30; 95% CI, 3.28–16.21, *p* < 0.001), and D-dimer >4.72 μg/ml (OR 4.64; 95% CI, 1.80–11.72, *p* = 0.001) were risk factors for the occurrence of IVCT (shown in Figure 3), and these five features (shown in Table 2) were used to establish the predictive model. The final model to predict the probability of IVCT is summarized as follows:


$$\begin{array}{l} \ln\;\left(\;\frac{\text{P}}{\text{1}-\text{P}}\;\right)=\text{0}.\text{930}\times \text{Age}+\text{1}.\text{038 }\times \text{Male}\\ +\text{2}.\text{105 }\times \text{Proximal LEDVT}+\text{1}.\text{987}\times \text{Bilateral LEDVT}\\ +\text{1}.\text{535 }\times \text{D}-\text{dimer}-\text{6}.\text{608} \end{array}$$

**Table 2:** Multivariable Model to Predict IVCT.

**Variables**	***B***	**OR (95% CI)**	***p-*value**
Age < 63.5 years	0.930	1.099–5.845	0.029
Male	1.038	1.187–6.719	0.019
Proximal LEDVT	2.105	1.010–66.764	0.049
Bilateral thrombus in limbs	1.987	3.280–16.211	0.000
D-dimer >4.72 μg/ml	1.535	1.800–11.724	0.001
Intercept	−6.608		

IVCT, inferior vena cava thrombosis; LEDVT, lower extremity deep vein thrombosis; B, regression coefficient; OR, odds ratio; CI, confidence interval.

where “*P*” is the probability of IVCT. The age, male sex, proximal LEDVT, bilateral LEDVT and D-dimer variables are deemed as 1 or 0, which are composed of the following: age < 63.5 years = 1, age > 63.5 years = 0; male = 1, female = 0; proximal LEDVT = 1, IDDVT = 0; bilateral LEDVT = 1, unilateral LEDVT = 0; D-dimer >4.72 μg/ml = 1, D-dimer < 4.72 μg/ml = 0.

The appropriateness of the fitted logistic regression model was examined by GOF using the Hosmer–Lemeshow GOF test (*p* = 0.883), which indicated good calibration and discriminative of the present predictive model. When using the ROC curves to identify the diagnostic efficiency, the AUC was 0.858 (95% CI, 0.799~0.916, *p* < 0.001) (shown in Figure 2). This predictive model had a sensitivity of 64.8% and a specificity of 90.0%. The positive predictive value for this value was 97.4%, and the negative predictive value was 31.0%. In predicting IVCT, the diagnostic efficiency of the multivariable model was significantly better than a single risk factor alone for age < 63.5 years (*p* < 0.001) or D-dimer >4.72 μg/ml (*p* < 0.001).

## Discussion

The occurrence of IVCT is related to the pathological and clinical spectrum of DVT (5); however, the predisposing risk factors for its formation remain uncommonly pursued and identified (2, 13). As IVCT can be seen as the consequence of thrombus propagation of LEDVT, it is essential to know whether the IVCT patients share LEDVT risk factors, predisposing disorders or if additional risk elements contribute to its development (3, 13). Therefore, raising awareness of IVCT, seeking the risk factors linked to it and subsequently establishing a reliable predictive model to predict its development may yield important clinical implications, which could be rushed into the forefront of clinicians. In the present study, completely based on demographics, presentation of LEDVT, laboratory examination, thrombus characteristics, comorbidities and risk factors for LEDVT, we found that bilateral LEDVT was associated with a higher incidence of IVCT than unilateral LEDVT, whereas the morbidity was not increased in any (left/right) extremity compared with the other one (right/left). IVCT was more prevalent among patients with proximal LEDVT than in IDDVT patients. Hence, the magnitude of risks for IVCT may be that bilateral LEDVT > left/right side LEDVT and proximal LEDVT > IDDVT. Age < 63.5 years, male sex, proximal LEDVT, bilateral LEDVT and D-dimer > 4.72 μg/ml were taken into account as risk factors when determining the occurrence of IVCT events. Furthermore, we derived a multivariable predictive model based on the above risk factors to predict the probability of developing IVCT among patients with LEDVT.

As previously reported, the incidence of IVCT is between 1.4 and 1.8 per 100,000 for the whole population (5) and 2.6–4.0% for patients with confirmed LEDVT, but this range may underestimate the real incidence because IVCT can be clinically silent and may become only revealed after sudden and fatal PE (8). In other studies (4, 6, 12, 14), it was reported that the lifetime incidence of LEDVT is 0.1% with a 4–15% incidence of IVCT, but given the number of unretrieved IVC filters, the incidence can reach 18.6%. Similarly, the incidence of nonfilter-associated IVCT among patients with LEDVT in this study was 15.0%, which is consistent with the prior literature (5). This high risk of developing IVCT may be partly attributable to a variety of confirmed IVCT patients who were referred from other hospitals, owing to their limited medical resources to deal with this intractable condition; thus, these may induce overestimated incidence, and caution should be taken in interpreting the incidence of nonfilter-associated IVCT in LEDVT. Although IVCT may be considered a subset of LEDVT and as such should share common etiologies, there are some aspects that require closer reflection (8, 15).

Although it has been shown that patients with LEDVT have increased morbidity from PE, few studies have focused on the relationship between the incidence of IVCT and PE as independent individuals. A prior study (7) showed that IVCT was a risk factor for silent or symptomatic PE, and LEDVT patients with IVC involvement had a relatively higher incidence of symptomatic PE than those with isolated lower extremity DVT (32.1 vs. 15.2%, *p* = 0.005), which seems to bear some resemblance to those in patients with simplex proximal LEDVT. In the present study, the patients with IVCT had a higher PE rate risk than patients with non-IVCT, but this did not reach statistical significance (62.5 vs. 54.2%, *p* = 0.329). Of note, the occurrence of PE mainly located in the proximal pulmonary arteries in IVCT patients was a higher rate risk than that in non-IVCT patients (32.0 vs. 16.3%, *p* = 0.067), implying that a non-negligible number of patients with IVCT will be more likely to encounter a potential risk of pulmonary hypertension and even life-threatening conditions, which was consistent with the prior findings that the mortality associated with IVCT is twice that of LEDVT alone (9); therefore, early detection of IVCT is of paramount importance because thrombi in the IVC can be floating and, if dislodged, can result in fatal PE (8). However, the morbidity reported in the present study may be influenced by some confounding variables linked to proximal or bilateral LEDVT, which are known to occur more frequently in cases of proximal or bilateral LEDVT (5, 8). Thus, further targeted studies may be needed to illustrate the relationship between PE and IVCT and proximal and bilateral LEDVT. Based on these potential risks, our study indicated that the decision-making process of aggressive treatment should be performed more carefully by evaluating the risk-benefit rate.

Although anticoagulation treatment remains a mainstay for IVCT, endovascular techniques aimed at restoring IVC patency have become key adjunctive therapies in recent years (16). The therapy modality for IVCT should take into account the efficacy of symptom relief as well as the risk of significant PE, which need protection measures prior to future surgeries. The aim of IVC filter interruption is to mechanically prevent massive venous thrombosis from reaching pulmonary circulation (17). However, the application of IVC filters for LEDVT is still controversial (18). The guideline considers that the indications for IVC filter placement in free-floating thrombi have not been confirmed, and broad use is based mainly on the perceived high risk of PE (17). PE was also significantly more aggressive in IVCT patients (19). Thus, under this condition, temporary filters are advised to be inserted into the IVC for patients with extensive thrombi in IVCT that are evaluated as potentially life-threatening prior to further treatments. If needed, filters in current use can be retrieved after several weeks or months to prevent long-term complications.

Through multivariate analysis, this study showed that patients aged < 63.5 years (sensitivity: 55.1%, specificity: 70.0%) had an 2.54-fold higher rate of IVCT than patients aged > 63.5 years, suggesting that younger patients have higher risk for IVCT. Similarly, other studies also found that a higher IVCT incidence was associated with age (19, 20). Inheritable thrombophilia is one of the main risk factors for IVCT, onset age of IVCT patients with inheritable thrombophilia is relatively very young (20). But in the present study the detection of inheritable thrombophilia was not completely performed in all patients. Therefore, a complete thrombophilia screen should be carried out in young patients with IVCT. Data regarding the effect of male sex on IVC filter thrombosis (OR, 5.0) were shown (20). In our study, the relationship between nonfilter-associated IVCT and male sex among LEDVT patients was also analyzed, and the results showed that male patients had an 2.82-fold higher rate of IVCT than female patients. Prior studies regarding LEDVT demonstrated that LEDVT can trigger increased IVCT risk with the extension of clot burden (2, 3, 10, 19). Of note, in most previous studies, the diagnosis of patients with LEDVT was only verified by duplex ultrasonography or direct venography (2, 5); thus, the detailed correlation of IVCT and thrombus characteristics of LEDVT has not been extensively elucidated. The present study showed that patients with a proximal LEDVT had an 8.21-fold increased risk for IVCT compared with patients with IDDVT, and the risk of IVCT was higher (7.29-fold) in bilateral LEDVT patients than in unilateral LEDVT patients, which suggested that CT angiography of the IVC performed in proximal or bilateral LEDVT patients to screen IVCT may be reasonable and meaningful. While a subgroup analysis was carried out in unilateral LEDVT patients, there was not a clear statistical significance in the difference between the left side and right side. Moreover, D-dimer testing was another risk factor associated with a high negative predictive value and a low positive predictive value for VTE diagnosis (7, 8). Our study demonstrated that LEDVT patients with IVCT had a significantly higher D-dimer level than those without, and we found that a D-dimer value of 4.72 μg/ml (sensitivity: 51.1%, specificity: 80.0%) was discriminative of IVCT occurrence. The multivariate regression model showed that a D-dimer value > 4.72 μg/ml was associated with an 4.64-fold increased risk of IVCT compared with a lower D-dimer value. Thus, screening for IVCT in LEDVT patients with a D-dimer value > 4.72 μg/ml might be reasonable. Aside from the factors mentioned above, external compression of the IVC by neighboring pathologic processes or left iliac vein compression may be other causes of IVCT development (20, 21). There are not enough included cases to warrant a significant difference in our cohort, but these factors may be worth studying in the future with large sample sizes.

According to the current pathophysiologic concept, several risk factors need to interact to trigger thrombosis (8, 15), which is not to assume that this concept does not hold true in IVCT patients. The risk factors mentioned above are likely to have complex interactions in fostering the occurrence of IVCT. The risk factors for IVCT tend to be multifaceted; these risk factors are speculative and cannot be tested to determine which factor might play the dominant role (8). Under this condition, we used multivariable logistic regression analysis to evaluate the contribution of risk factors for IVCT patients and established a multivariable predictive model. The AUC of the ROC curve for it was 0.858 and was with GOF using the Hosmer–Lemeshow GOF test (*p* = 0.883), which indicated significant predictive value of the present prediction model. In addition, the ROC curve showed that the diagnostic efficiency of this predictive model for predicting IVCT was superior to that of a single risk factor alone, including age < 63.5 years (AUC of ROC curve was 0.624) or D-dimer > 4.72 μg/ml (AUC of ROC curve was 0.656). Hence, we believe these factors may play an important role in predicting IVCT in patients with LEDVT.

The present study has several limitations that merit discussion. First, this was a single-center retrospective cohort study and not a population-based study or nationwide survey, therefore, it has inherent limitations of selection bias and reporting bias. Second, the sample sizes included in this study were relatively small for assessing the risk factors and establishing the multivariate predictive model. Extended sample size and external validation in multiple centers was missing; therefore, this model should be interpreted with caution. Hopefully, this model will be further validated in a large, multicenter, prospective validation study before providing benefits through its use. Third, the IVC anomaly may also expose patients to the risk of thrombosis (2); however, a thrombus may hide its detection. Finally, some patients underwent anticoagulation treatment before the blood sample was collected, which may have influenced the results. In the future, a study including more factors and excluding confounding factors to overcome these limitations can be designed to further improve and refine this model.

## Conclusion

This study showed that age < 63.5 years, male sex, proximal LEDVT, bilateral LEDVT and D-dimer > 4.72 μg/ml were risk factors for the occurrence of IVCT, which suggests that intense surveillance for these patients is essential because of the relatively high IVCT occurrence in the population. The diagnostic efficiency of the multivariable predictive model was superior to that of a single risk factor, such as age < 63.5 years or D-dimer > 4.7 2 μg/ml. Hence, we believe that it is likely to be a promising method for evaluating and predicting the probability of IVCT in confirmed LEDVT patients.

## Data availability statement

The original contributions presented in the study are included in the article/supplementary material, further inquiries can be directed to the corresponding authors.

## Ethics statement

The studies involving human participants were reviewed and approved by Nanjing First Hospital, Nanjing Medical University (Nanjing, China). The patients/participants provided their written informed consent to participate in this study.

## Author contributions

MG contributed to this project development and manuscript writing/editing. JK and YS contributed to data collection and manuscript-associated editing. JK contributed to the data analysis. BZ and ZL contributed to manuscript editing. XH and JG contributed to project development and manuscript editing. All authors contributed to the article and approved the submitted version.

## References

[1] Kakkos SK Gohel M Baekgaard N Bauersachs R Bellmunt-Montoya S Black SA. Editor's choice-European society for vascular surgery (ESVS) 2021 clinical practice guidelines on the management of venous thrombosis. Eur J Vasc Endovasc Surg. (2021) 61:9–82. 10.1016/j.ejvs.2020.09.023 33334670

[2] Alkhouli M Morad M Narins CR Raza F Bashir R. Inferior vena cava thrombosis. JACC Cardiovasc Interv. (2016) 9:629–43. 10.1016/j.jcin.2015.12.268 26952909

[3] Avgerinos ED El-Shazly O Jeyabalan G Al-Khoury G Hager E Singh MJ. Impact of inferior vena cava thrombus extension on thrombolysis for acute ilio-femoral thrombosis. J Vasc Surg Venous Lymphat Disord. (2016) 4:385–91. 10.1016/j.jvsv.2016.05.005 27638990

[4] Stein PD Matta F Yaekoub AY. Incidence of vena cava thrombosis in the United States. Am J Cardiol. (2008) 102:927–9. 10.1016/j.amjcard.2008.05.046 18805124

[5] McAree BJ O'Donnell ME Fitzmaurice GJ Reid JA Spence RA Lee B. Inferior vena cava thrombosis: a review of current practice. Vasc Med. (2013) 18:32–43. 10.1177/1358863X12471967 23439778

[6] Agnelli G Verso M Ageno W Imberti D Moia M Palareti G. The MASTER registry on venous thromboembolism: description of the study cohort. Thromb Res. (2008) 121:605–10. 10.1016/j.thromres.2007.06.009 17692901

[7] Shi Y Wang T Yuan Y Su H Chen L Huang H. Silent pulmonary embolism in deep vein thrombosis: relationship and risk factors. Clin Appl Thromb Hemost. (2022) 28:10760296221131034. 10.1177/10760296221131034 36199255 PMC9537479

[8] Shi W Dowell JD. Etiology and treatment of acute inferior vena cava thrombosis. Thromb Res. (2017) 149:9–16. 10.1016/j.thromres.2016.07.010 27865097

[9] Hanterdsith B. Fatal pulmonary thromboembolism due to inferior vena cava thrombosis. Ann Vasc Dis. (2011) 4:121–3. 10.3400/avd.cr.10.00021 23555441 PMC3595832

[10] Kraft C Hecking C Schwonberg J Schindewolf M Lindhoff-Last E Linnemann B. Patients with inferior vena cava thrombosis frequently present with lower back pain and bilateral lower-extremity deep vein thrombosis. Vasa. (2013) 42:275–83. 10.1024/0301-1526/a000288 23823859

[11] Purvis JA Campbell DM McCarron MO. Embolic stroke as a late complication of inferior vena cava thrombosis. Ulster Med J. (2010) 79:30. 20844730 PMC2938997

[12] Giordano P Weber K Davis M Carter E. Acute thrombosis of the inferior vena cava. Am J Emerg Med. (2006) 24:640–2. 10.1016/j.ajem.2005.12.018 16938618

[13] Linnemann B Schmidt H Schindewolf M Erbe M Zgouras D Grossmann R. Etiology and VTE risk factor distribution in patients with inferior vena cava thrombosis. Thromb Res. (2008) 123:72–8. 10.1016/j.thromres.2008.01.004 18295303

[14] White RH. The epidemiology of venous thromboembolism. Circulation. (2003) 107(23 Suppl 1):14–8. 10.1161/01.CIR.0000078468.11849.66 12814979

[15] Xiao N Karp J Lewandowski R Cuttica M Schimmel D Martin K. Inferior vena cava thrombosis risk in 1,582 patients with inferior vena cava filters. Radiology. (2022) 303:300–2. 10.1148/radiol.211169 35133197

[16] Ye K Qin J Yin M Liu X Lu X. Outcomes of pharmacomechanical catheter-directed thrombolysis for acute and subacute inferior vena cava thrombosis: a retrospective evaluation in a single institution. Eur J Vasc Endovasc Surg. (2017) 54:504–12. 10.1016/j.ejvs.2017.06.025 28801136

[17] Cohen O Ageno W Farjat AE Turpie AGG Weitz JI Haas S. Management strategies and clinical outcomes in patients with inferior vena cava thrombosis: data from GARFIELD-VTE. J Thromb Haemost. (2022) 20:366–74. 10.1111/jth.15574 34714962 PMC9299483

[18] Ahmad I Yeddula K Wicky S Kalva SP. Clinical sequelae of thrombus in an inferior vena cava filter. Cardiovasc Intervent Radiol. (2010) 33:285e9. 10.1007/s00270-009-9664-x 19688372

[19] Seinturier C Bosson JL Colonna M Imbert B Carpentier PH. Site and clinical outcome of deep vein thrombosis of the lower limbs: an epidemiological study. J Thromb Haemost. (2005) 3:1362–7. 10.1111/j.1538-7836.2005.01393.x 15892854

[20] Liu XR Zhou W Chen F. Severe compression of left iliac vein is a protective factor for the risk of inferior vena cava thrombosis. J Vasc Surg Venous Lymphat Disord. (2022) 10:1107–12. 10.1016/j.jvsv.2022.04.018 35716997

[21] Teter K Schrem E Ranganath N Adelman M Berger J Sussman R. Presentation and management of inferior vena cava thrombosis. Ann Vasc Surg. (2019) 56:17–23. 10.1016/j.avsg.2018.08.082 30982504

